# Safety of two-dose schedule of COVID-19 adsorbed inactivated vaccine (CoronaVac; Sinovac/Butantan) and heterologous additional doses of mRNA BNT162b2 (Pfizer/BioNTech) in immunocompromised and immunocompetent individuals

**DOI:** 10.1590/S1678-9946202567002

**Published:** 2025-01-20

**Authors:** Karina Takesaki Miyaji, Karim Yaqub Ibrahim, Vanessa Infante, Raquel Megale Moreira, Carolina Ferreira dos Santos, Juliana de Cássia Belizário, Maria Isabel de Moraes Pinto, Ana Karolina Barreto Berselli Marinho, Juliana Marquezi Pereira, Liliane Saraiva de Mello, Vitor Gabriel Lopes da Silva, Paula Keiko Sato, Tânia Mara Varejão Strabelli, Lucas Ragiotto, Pedro Henrique Theotonio de Mesquita Pacheco, Patricia Emilia Braga, Ana Paula Loch, Alexander Roberto Precioso, Ana Marli Christovam Sartori, Study Group, João Ítalo França, Marcos Alves de Lima, Mauricio Cesar Sampaio Ando, Camila Cristina Martini Rodrigues, Alice Tung Wan Song, Amanda Nazareth Lara, Ana Cristina Belizário, Anna Helena Simões Bortulucci de Lima, Ariane Cristina Barboza Zanetti, Audrey Rose da Silveira Amancio de Paulo, Bruna Del Guerra de Carvalho Moraes, Bruna Ribeiro de Oliveira, Camila de Melo Picone, Carolina Sanches Aranda, Carolinne Paioli Troli, Cristina M. Kokron, Debora Raquel Benedita Terrabuio, Edson Abdala, Elias David, Érika Yoshie Shimoda Nakanishi, Fabiana Mascarenhas Souza Lima, Fabio Batista Firmino, Fernanda Barone Alves dos Santos, Fernando Bacal, Giancarlo Fatobene, Jaqueline Oliveira Santana, Jorge Kalil, Julia Barbosa, Leandro Peres Gonçalves, Leonardo Jun Otuyama, Ligia Camera Pierrotti, Livia Caroline Mariano Compte, Livia Netto Chaer, Luis Fernando Seguro, Luiz Sergio Azevedo, Márcia Aiko Ueda, Maria Teresa Terreri, Myrthes Anna Maragna Toledo Barros, Octávio Grecco, Odeli Nicole Encinas Sejas, Priscila Tavares Musqueira, Raquel Keiko de Luca Ito, Samia Silveira Souza Teixeira, Serafim Fidalgo, Silvia Figueiredo Costa, Silvia Vidal Campos, Tamiris Hinsching Fernandes, Vanderson Geraldo Rocha, Vivian Caso Coelho

**Affiliations:** 1 Universidade de São Paulo, Faculdade de Medicina, Hospital das Clínicas, Divisão de Moléstias Infecciosas e Parasitarias, São Paulo, São Paulo, Brazil; 2 Universidade de São Paulo, Faculdade de Medicina, Hospital das Clínicas, Centro de Referência para Imunobiológicos Especiais, São Paulo, São Paulo, Brazil; 3 Instituto Butantan, Divisão de Ensaios Clínicos e Farmacovigilância, Centro de Farmacovigilância, Segurança Clínica e Gestão de Risco, São Paulo, São Paulo, Brazil; 4 Universidade de São Paulo, Faculdade de Medicina, Instituto do Câncer do Estado de São Paulo, Serviço de Controle de Infecção Hospitalar, São Paulo, São Paulo, Brazil; 5 Universidade de São Paulo, Faculdade de Medicina, Hospital das Clínicas, Serviço de Transplante Renal, SSão Paulo, São Paulo, Brazil; 6 Universidade de São Paulo, Faculdade de Medicina, Hospital das Clínicas, Divisão de Clínica Médica, Serviço de Hematologia, Hemoterapia e Terapia Celular, São Paulo, São Paulo, Brazil; 7 Universidade Federal de São Paulo, Escola Paulista de Medicina, Departamento de Pediatria, Disciplina de Alergia, Imunologia Clínica e Reumatologia, São Paulo, São Paulo, Brazil; 8 Universidade de São Paulo, Faculdade de Medicina, Hospital das Clínicas, Divisão de Imunologia Clínica, Departamento de Clínica Médica, São Paulo, São Paulo, Brazil; 9 Universidade de São Paulo, Faculdade de Medicina, Hospital das Clínicas, Divisão de Transplante de Fígado e Órgãos do Aparelho Digestivo, São Paulo, São Paulo, Brazil; 10 Universidade de São Paulo, Faculdade de Medicina, Instituto do Coração, Serviço de Pneumologia, Unidade de Transplante de Pulmão, São Paulo, São Paulo, Brazil; 11 Universidade Federal de São Paulo, Disciplina de Infectologia Pediátrica, Laboratório de Pesquisas, São Paulo, São Paulo, Brazil; 12 Universidade de São Paulo, Faculdade de Medicina, Hospital das Clínicas, Divisão de Clínica de Moléstias Infecciosas e Parasitárias, Laboratório de Investigação Médica em Imunologia (LIM-48), SSão Paulo, São Paulo, Brazil; 13 Universidade de São Paulo, Faculdade de Medicina, Hospital das Clinicas, Instituto do Coração, Subcomissão de Controle de Infecção Hospitalar, São Paulo, São Paulo, Brazil; 14 Universidade de São Paulo, Faculdade de Medicina, Departamento de Infectologia e Medicina Tropical, São Paulo, São Paulo, Brazil; 15 Instituto Butantan, Laboratório Estratégico de Diagnóstico, São Paulo, São Paulo, Brazil; 16 Universidade de São Paulo, Faculdade de Medicina, Hospital das Clinicas, Instituto do Coração, Serviço de Cardiologia, Unidade de Transplante de Coração, São Paulo, São Paulo, Brazil

**Keywords:** COVID-19 vaccines, BNT162 vaccine, Inactivated vaccine, Immunocompromised host, Safety

## Abstract

Immunocompromised individuals were considered high-risk for severe disease due to SARS COV-2 infection. This study aimed to describe the safety of two doses of COVID-19 adsorbed inactivated vaccine (CoronaVac; Sinovac/Butantan), followed by additional doses of mRNA BNT162b2 (Pfizer/BioNTech) in immunocompromised (IC) adults, compared to immunocompetent/healthy (H) individuals. This phase 4, multicenter, open label study included solid organ transplant and hematopoietic stem cell transplant recipients, cancer patients and people with inborn errors of immunity with defects in antibody production, rheumatic, end-stage chronic kidney or liver disease, who were enrolled in the IC group. Participants received two doses of CoronaVac and additional doses of mRNA BNT162b2. Adverse reactions (AR) data were collected within seven days after each vaccination. Serious adverse events and of special interest (AESI) were monitored throughout the study. We included 241 immunocompromised and 100 immunocompetent subjects. Arthralgia, fatigue, myalgia, and nausea were more frequent in the IC group after CoronaVac. Following the first additional dose of mRNA BNT162, pain, induration, and tenderness at injection site, fatigue and myalgia were more frequent in the H group. A heart transplant recipient had a graft rejection temporally associated with the second CoronaVac dose, but there was no literature evidence of causal association. Four cases of AESI were considered related to the vaccine: three erythema multiforme after CoronaVac, all in IC participants, and one paresthesia after mRNA, in a H participant. Our findings were comparable to other studies that evaluated the safety of COVID-19 vaccines in different immunocompromised populations. Both vaccines were safe for immunocompromised participants.

## INTRODUCTION

Great scientific efforts were made to develop effective and safe COVID-19 vaccines, whose rapid development benefited from the previously studied SARS-CoV-1 and MERS-CoV vaccines^1^, as well as from parallel phase 1 and 2 and parallel phase 2 and 3 trials. Both traditional platforms, such as inactivated virus vaccines, and new platforms, such as nonreplicating viral vectors (adenovirus), nucleic acids (DNA and mRNA), and virus-like particles (VLP) were used, and several vaccine candidates reached clinical stage in less than six months. Due to those efforts and substantial financial investments, some vaccines were approved for emergency use, and vaccination began, in many countries, in December 2020.

At first, two vaccines were approved by the Brazilian Regulatory Agency (ANVISA): the inactivated virus vaccine (CoronaVac) developed by Sinovac Life Science Co., which had a technology transfer agreement with Instituto Butantan, and the nonreplicating viral vector vaccine with the spike (S) protein gene (chimpanzee Adenovirus, ChAdOx), from Oxford University/AstraZeneca, which had a technology transfer agreement with Bio-Manguinhos^2^. Later, two other vaccines, the nonreplicating viral vector (Adenovirus 26) from Janssen^3^, and the mRNA encoding the RBD portion of the spike protein wrapped in a lipid nanoparticle, (BNT162b2) by Pfizer/BioNTech, were granted with definitive registry^4^. Vaccination began in Brazil on January 16^th^ 2021, prioritizing healthcare workers, immunocompromised individuals and older adults, increasingly reaching younger age groups, depending on the availability of doses. In September 2021, the Brazilian Ministry of Health recommended a third dose for all immunocompromised individuals and, progressively, for the healthy population. The fourth dose, administered four months after the last one, was recommended for immunocompromised individuals in December 2021. This population was not included in clinical trials, so data on safety, immunogenicity and effectiveness of COVID-19 vaccines for this population come from phase 4 studies. Few studies evaluated the safety of CoronaVac for immunocompromised persons. Two studies included HIV infected people who received CoronaVac as primary vaccination and compared them to immunocompetent people^5,6^. Mild to moderate adverse reactions were reported and no serious cases were reported. Another study included patients with autoimmune rheumatic diseases and no serious/moderate adverse reactions were detected^7^.

This study aimed to describe the safety of a two-dose schedule of the COVID-19 adsorbed inactivated vaccine (CoronaVac), followed by two additional doses of mRNA in immunocompromised adults, compared to two doses of CoronaVac and a booster of BNT162b2 in immunocompetent individuals.

## MATERIALS AND METHODS

This was a phase 4, multicenter, open-label trial to evaluate the safety and immunogenicity of COVID-19 vaccines in immunocompromised adults compared to immunocompetent adults. The study was conducted at the Hospital das Clinicas (HC-FMUSP), Instituto do Coracao (INCOR-FMUSP), Instituto do Cancer de Sao Paulo (ICESP) and Hospital Sao Paulo (HSP-UNIFESP), in Sao Paulo city, Brazil. Participants were enrolled from May 28^th^ to October 6^th^, 2021.

### Study population

Detailed description of the study population and procedures was previously reported^8^. Results of immunogenicity of COVID-19 adsorbed inactivated vaccine (CoronaVac) and additional doses of mRNA BNT162b2 in these participants were previously published^8^.

Briefly, the immunocompromised group comprised adults aged ≥18 years with solid organ transplant (SOT, liver, kidney, lung and heart); hematopoietic stem cell transplant (HSCT); solid organ and hematological malignancies; inborn errors of immunity with defects in antibody production; immune-mediated rheumatic diseases, and end-stage chronic kidney or liver disease waiting for transplantation. The comparison group included immunocompetent adults aged ≥18 years.

### COVID-19 vaccination

All participants received two doses of CoronaVac, with a 28-day interval between doses. CoronaVac doses were provided by Instituto Butantan. Immunocompromised participants received two additional doses: a third at least 28 days after the second dose and a fourth at least four months after the third one, following the Brazilian Ministry of Health (MoH) recommendations. Immunocompetent persons received a third dose at least four months after the primary schedule. Additional doses were provided by MoH. Most participants received BNT162b2 mRNA vaccine as additional doses. The few participants who received a vaccine other than BNT162b2 mRNA as additional doses were excluded from analyses, except in serious adverse events and adverse events of special interest, in which all were described. Vaccine batches used in the study are described in Supplementary File S1.

### Safety data collection

All participants received a diary to register local and systemic adverse events (AE) that occurred within seven days after each vaccination. AE and COVID-19 symptoms and diagnosis were monitored by phone calls, text messages (SMS, WhatsApp) or email 10-15 days after each vaccine dose and monthly throughout the study period (four to six months after the last dose). Local solicited AE included: pain, tenderness, edema, erythema, induration, and pruritus. Solicited systemic AE included: fever, malaise, fatigue, myalgia, arthralgia, chills, nausea, vomiting, diarrhea, anorexia, rash, pruritus, cough, and allergic reactions.

Serious adverse events (SAE) and adverse events of special interest (AESI)^9^ were monitored throughout the study period.

### Statistical analysis

Study data were collected and managed using REDCap^®^ (Vanderbilt University) electronic data capture tools hosted at Hospital das Clinicas.

A descriptive analysis of adverse reactions (AR), that is, any vaccine-related adverse event occurring within seven days, was conducted. Solicited, unsolicited, local and systemic AR were described according to vaccine dose and participants’ group. AESI and SAE were described according to vaccine dose and participants’ group during the study period. Events were listed individually for each participant and summarized according to frequency, intensity and duration according to vaccine dose and participants’ group.

Demographic characteristics were compared using Mann-Whitney test for continuous variables and Fisher’s exact test for categorical variables. Safety analysis was performed for all AR by vaccine dose and group. Fisher’s exact test was used for comparisons between groups. Moreover, McNemar’s test was used to compare the number of participants with AR after CoronaVac (first and second doses) and mRNA BNT162b2 (third and fourth doses). Percentages were calculated with their respective 95% CI by Clopper-Pearson method. All statistical tests were two-sided, with *p*<0.05 adopted for statistical significance. Analyses were conducted using R.

### Ethical issues

The original protocol and all changes made during the study conduction were approved by the Research Ethics Committees of the participating institutions and the National Research Ethics Committee (CONEP, CAAE Nº 87498318.0.0000.0068). The protocol was registered at the Brazilian Registry of Clinical Trials (REBEC, RBR-9ksh5f4). All participants provided written informed consent before enrollment. Participants identification remained confidential throughout the study and analyses.

## RESULTS

**Figure 1:**
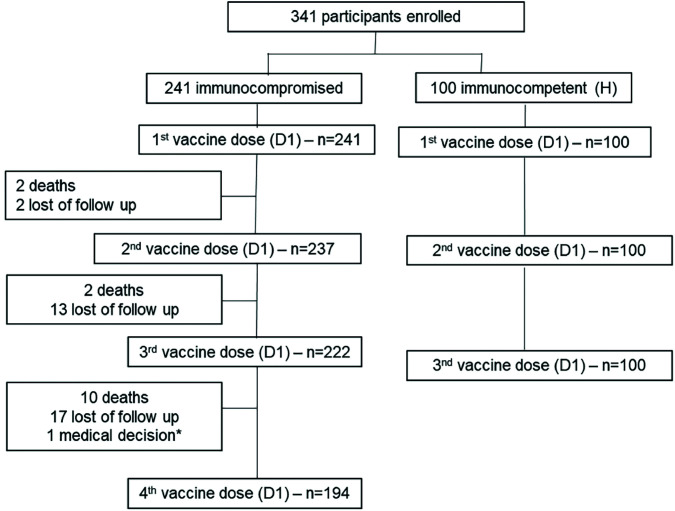
Flowchart of study population. *Participant was hospitalized for a long period due to cancer complications.

From May 28^th^ to October 6^th^, 2021, 341 participants were enrolled in the study: 241 immunocompromised subjects (114 SOT recipients, 30 HSCT recipients, 27 cancer patients, 44 individuals with inborn errors of immunity [IEI], 21 with rheumatic diseases and five with end-stage chronic diseases pre-transplantation) and 100 immunocompetent subjects (Figure 1).

Demographic characteristics of immunocompromised (IC) and immunocompetent (H) participants were similar (Table 1), except for schooling, which was higher in the H group than in the IC group (median, 15 and 11 years, respectively, *p*<0·001). The median age was 36 in the IC group and 37 in the H group. There were 129 (53.5%) and 48 (48% ) women in the IC and group, respectively.

**Table 1:** - Demographic characteristics of participants enrolled in the phase 4 study of COVID-19 vaccines safety, according to group.

	Immunocompromised (n=241)		Immunocompetent (n=100)		*p*-value^**a**^
**Age (years)**					
Median (Q_1_-Q_3_)	36.0	(26.0 - 50.0)	37.0	(31.0 - 44.0)	0.992
**Sex, n (%)**					0.405
Female	129	(53.5)	48	(48.0)	
Male	112	(46.5)	52	(52.0)	
**Color, n (%)**					0.663
White	135	(56.0)	59	(59.0)	
Non-white	106	(44.0)	41	(41.0)	
**Educational level (years)**					**<0.001**
Median (Q_1_-Q_3_)	11	(9 - 12)	15	(11 - 16)	
**Immunosuppressors and disease-modifying anti-rheumatic drugs** ^b^ **n (%)**					
Prednisone	126	(52.3)			
Tacrolimus	100	(41.5)			
Mycophenolate	93	(38.6)			
Ciclosporin	21	(8.7)			
Azathioprine	16	(6.6)			
Methotrexate	12	(5.0)			
Sirolimus	8	(3.3)			
Everolimus	7	(2.9)			
Leflunomide	2	(0.8)			
Sulfasalazine	2	(0.8)			
Other	24	(10.0)			

Q1 = first quartile; Q3 = third quartile; ^a^Mann-Whitney test; ^b^Only for the immunocompromised group.

All 341 participants received the first CoronaVac dose; 237 IC and all 100 H received the second CoronaVac dose; 222 IC and 100 H received the third mRNA BNT162b2 vaccine; and 194 immunocompromised participants received the fourth mRNA BNT162b2 dose (Figure 1).

Table 2 shows the number of adverse reactions (AR) after each dose and the number of participants with at least one AR following vaccination, according to the study group. AR was more frequent in the H group when compared to the IC group, but statistical significance was obtained only after the third dose (*p*<0.001). The frequency of AR following CoronaVac (first and second doses) and mRNA BNT162b2 (third and fourth doses) were similar in both the IC group (*p*=0.201) and the H group (*p*=0.435).

**Table 2:** - Number of participants with at least one local or systemic adverse reactions (AR) after each COVID-19 vaccine dose and comparison between CoronaVac (first and second doses) and mRNA BNT162b2 (third and fourth doses), by study group.

Vaccine dose	Study group		*p*-value^b^
	Immunocompromised	Immunocompetent^a^	
**First dose (CoronaVac)**			
AR (n)	580	168	
Participants with AR, n / Vaccinated, n (%)	152 / 241 (63.1)	69 / 100 (69.0)	0.321
AR per participant, Median (Q1-Q3)	3 (1 - 5)	2 (1 - 3)	
**Second dose (CoronaVac)**			
AR (n)	375	134	
Participants with AR, n / Vaccinated, n (%)	113 / 237 (47.7)	55 / 100 (55.0)	0.235
AR per participant: Median (Q1-Q3)	3 (1 - 4)	2 (1 - 3)	
**Third dose (mRNA BNT162b2)**			
AR (n)	628	362	
Participants with AR, n/ Vaccinated, n (%)	149 / 222 (67.1)	86 / 100 (86.0)	**<0.001**
AR per participant, Median (Q1-Q3)	3 (2 - 6)	3 (2 - 6)	
**Fourth dose (mRNA BNT162b2**)			
AR (n)	581	–	
Participants with AR, n/ Vaccinated, n (%)	114 / 194 (58.8)	–	
AR per participant, Median (Q1-Q3)	4 (2 - 8)	–	
**CoronaVac (first and second) doses**			
AR (n)	812	302	
Participants with AR (%)	141 (74.2)	79 (79.0)	
**mRNA BNT162b2 (third and fourth) doses**			
AR (n)	1153	362	
Participants with AR (%)	144 (75.8)	86 (86.0)	
*p*-value^c^	0.201	0.435	

^a^Immunocompetent received only three COVID vaccine doses during the study period; ^b^Fisher’s exact test; ^c^ *p*: McNemar’s test comparing number of participants with AR after CoronaVac (first and second dose) and after mRNA BNT162 (third and fourth dose).

Table 3 presents local and systemic AR after each dose in both immunocompromised and immunocompetent groups. After the first CoronaVac dose, arthralgia (*p*=0.012), fatigue (*p*=0.038), myalgia (*p*<0.001), and nausea (*p*=0.012) were significantly more frequent in the IC group. After the second CoronaVac dose, arthralgia (*p*=0.047) and myalgia (*p*=0.001) were more frequent in the IC group. After the third dose (BNT162b2), pain (*p*=0.015), induration (*p*=0.032) and tenderness (*p*=0.001) at injection site and fatigue (*p*=0.018) and myalgia (*p*<0.001) were significantly more frequent in the H group (Table 3). After the fourth dose (BNT162b2) in the IC group, the most frequent AR were pain (52.1%) and tenderness (32.5%) at injection site and headache (28.9%) (Table 3). Most solicited adverse reactions were mild to moderate in both groups (Supplementary Tables S1A, S1B, S1C and S1D). Non-solicited axillar pain (*p*=0.029) was also more frequent in the H group (Supplementary Tables S2A, S2B, S2C and S2D).

**Table 3:** Number of participants with local and systemic solicited adverse reactions (AR) after CoronaVac and mRNA BNT162b2, by dose, event and study group.

Adverse Reaction	First dose (CoronaVac)							Second dose (CoronaVac)							Third dose (mRNA BNT162b2)							Fourth dose (mRNA BNT162b2)		
	IC			H			*p*-value^**a**^	IC			H			*p*-value^**a**^	IC			H			*p*-value^**a**^	IC		
	(n=241)			(n=100)				(n=237)			(n=100)				(n=222)			(n=100)				(n=194)		
	n	%	(95%CI)	n	%	(95% CI)		n	%	(95% CI)	n	%	(95% CI)		n	%	(95% CI)	n	%	(95% CI)		n	%	(95% CI)
**Local**																								
Pain	102	42.3	(36.0-48.8)	40	40.0	(30.3-50.3)	0.719	77	32.5	(26.6-38.9)	36	36.0	(26.6-46.2)	0.531	139	62.6	(55.9-69.0)	77	77.0	(67.5-84.8)	**0.015**	101	52.1	(44.8-59.3)
Erythema	3	1.2	(0.3-3.6)	1	1.0	(0.0-5.4)	>0.999	1	0.4	(0.0-2.3)	1	1.0	(0.0-5.4)	0.506	16	7.2	(4.2-11.4)	8	8.0	(3.5-15.2)	0.820	14	7.2	(4.0-11.8)
Edema	9	3.7	(1.7-7.0)	0	0.0	(0.0-3.6)	0.063	5	2.1	(0.7-4.9)	0	0.0	(0.0-3.6)	0.327	30	13.5	(9.3-18.7)	19	19.0	(11.8-28.1)	0.241	31	16.0	(11.1-21.9)
Induration	7	2.9	(1.2-5.9)	0	0.0	(0.0-3.6)	0.111	3	1.3	(0.3-3.7)	2	2.0	0.2-7.0)	0.635	14	6.3	(3.5-10.4)	14	14.0	(7.9-22.4)	**0.032**	15	7.7	(4.4-12.4)
Pruritus	6	2.5	(0.9-5.3)	2	2.0	(0.2-7.0)	>0.999	8	3.4	(1.5-6.5)	2	2.0	(0.2-7.0)	0.729	10	4.5	(2.2-8.1)	5	5.0	(1.6-11.3)	0.784	15	7.7	(4.4-12.4)
Tenderness	60	24.9	(19.6-30.9)	34	34.0	(24.8-44.2)	0.110	40	16.9	(12.3-22.3)	23	23.0	(15.2-32.5)	0.221	73	32.9	(26.7-39.5)	54	54.0	(43.7-64.0)	**0.001**	63	32.5	(25.9-39.6)
**Systemic**																								
Decreased appetite	8	3.3	(1.4-6.4)	0	0.0	(0.0-3.6)	0.111	5	2.1	(0.7-4.9)	0	0.0	(0.0-3.6)	0.327	14	6.3	(3.5-10.4)	4	4.0	(1.1-9.9)	0.601	18	9.3	(5.6-14.3)
Arthralgia	28	11.6	(7.9-16.4)	3	3.0	(0.6-8.5)	**0.012**	14	5.9	(3.3-9.7)	1	1.0	(0.0-5.4)	**0.047**	19	8.6	(5.2-13.0)	16	16.0	(9.4-24.7)	0.054	22	11.3	(7.2-16.7)
Chills	24	10.0	(6.5-14.5)	7	7.0	(2.9-13.9)	0.535	17	7.2	(4.2-11.2)	3	3.0	(0.6-8.5)	0.206	25	11.3	(7.4-16.2)	15	15.0	(8.6-23.5)	0.364	30	15.5	(10.7-21.3)
Headache	75	31.1	(25.3-37.4)	26	26.0	(17.7-35.7)	0.365	46	19.4	(14.6-25.0)	17	17.0	(10.2-25.8)	0.649	60	27.0	(21.3-33.4)	33	33.0	(23.9-43.1)	0.290	56	28.9	(22.6-35.8)
Diarrhea	15	6.2	(3.5-10.1)	5	5.0	(1.6-11.3)	0.803	10	4.2	2.0-7.6)	1	1.0	(0.0-5.4)	0.185	15	6.8	(3.8-10.9)	6	6.0	(2.2-12.6)	>0.999	11	5.7	(2.9-9.9)
Rash	5	2.1	(0.7-4.8)	1	1.0	(0.0-5.4)	0.675	1	0.4	(0.0-2.3)	1	1.0	(0.0-5.4)	0.506	1	0.5	(0.0-2.5)	2	2.0	(0.2-7.0)	0.229	0	0.0	(0.0-1.9)
Fatigue	48	19.4	(15.125.5)	11	11.0	(5.6 – 18.8)	**0.038**	22	9.3	(5.9-13.7)	10	10.0	(4.9-17.6)	0.837	38	17.1	(12.4-22.7)	29	29.0	(20.4-38.9)	**0.018**	36	18.6	(13.3-24.8)
Hypersensitivity	5	2.1	(0.7-4.8)	0	0.0	(0.0-3.6)	0.327	2	0.8	(0.1-3.0)	0	0.0	(0.0-3.6)	>0.999	0	0.0	(0.0-1.6)	0	0.0	(0.0-3.6)	>0.999	2	1.0	(0.1-3.7)
Malaise	0	0.0	(0.0-1.5)	0	0.0	(0.0-3.6)	>0.999	1	0.4	(0.0-2.3)	0	0.0	(0.0-3.6)	>0.999	2	0.9	(0.1-3.2)	2	2.0	(0.2-7.0)	0.591	0	0.0	(0.0-1.9)
Myalgia	34	14.1	(10-19.2)	6	6.0	(2.2 – 12.6)	**<0.001**	26	11.0	(7.3 – 15.7)	6	6.0	(2.2 – 12.6)	**0.001**	43	19.4	(14.4 – 25.2)	27	27.0	(18.6 – 36.8)	**<0.001**	51	26.3	(20.2 – 33.1)
Nausea	23	9.5	(6.1-14.0)	2	2.0	(0.2-7.0)	**0.012**	18	7.6	(4.6-11.7)	3	3.0	(0.6-.5)	0.141	19	8.6	(5.2-13.0)	10	10.0	(4.9-17.6)	0.678	26	13.4	(8.9-19.0)
Fever	9	3.7	(1.7-7.0)	0	0.0	(0.0-3.6)	0.063	7	3.0	(1.2-6.0)	0	0.0	(0.0-3.6)	0.109	17	7.7	(4.5-12.0)	8	8.0	(3.5-15.2)	>0.999	20	10.3	(6.4-15.5)
Pruritus^b^	16	6.6	(3.8-10.6)	4	4.0	(1.1-9.9)	0.452	8	3.4	(1.5-6.5)	2	2.0	(0.2-7.0)	0.729	6	2.7	(1.0-5.8)	7	7.0	(2.9-13.9)	0.121	7	3.6	(1.5-7.3)
Cough	18	7.5	(4.5-11.5)	7	7.0	(2.9-13.9)	>0.999	10	4.2	(2.0-7.6)	4	4.0	(1.1-9.9)	>0.999	10	4.5	(2.2-8.1)	1	1.0	(0.0-5.4)	0.183	9	4.6	(2.1-8.6)
Vomiting	6	2.5	(0.9-5.3)	0	0.0	(0.0-3.6)	0.186	4	1.7	(0.5-4.3)	1	1.0	(0.0-5.4)	>0.999	7	3.2	(1.3-6.4)	0	0.0	(0.0-3.6)	0.104	6	3.1	(1.1-6.6)

^a^Fisher’s exact test; ^b^Other than vaccination site; IC = immunocompromise; H = immunocompetent/healthy.

Throughout the study, 110 SAE were reported in 63 participants (Table 4 and Supplementary Table S3). There were two cases of nephrolithiasis in two healthy participants (H), who needed hospitalizations. There were 13 hospitalizations due to COVID-19, all in the IC group: three after the first dose, two after the second dose, seven after the third dose and one after the fourth dose, and four participants died. Other 95 SAE occurred in 48 IC participants, with 46 hospitalizations and 15 deaths (Table 5). Among IC participants, most SAE were related to underlying conditions. Only one SAE was considered unexpected and possibly related to the vaccine. A heart transplant recipient had a graft rejection, whose symptoms (fever, dyspnea and nausea) started 11 days after the second CoronaVac dose. The participant was under immunosuppressive therapy but had two previous episodes of rejection, the last one two years before COVID-19 vaccination. He was hospitalized and fully recovered. Since no other cause was identified for this type of rejection, it was classified as B1 (temporal association to vaccination, but without evidence from literature to establish causal association). All remaining SAE were considered not related to the vaccines, according to the study’s physicians. Table 5 describes all deaths occurred during the study, and none was considered related to vaccine. Among them, 84.2% participants died over 30 days after vaccination. Cause of death was well established for three participants who died less than 30 days after vaccination (one from COVID-19 and two from progression of underlying cancer) and connection with the vaccine was not considered plausible.

**Table 4:** - Serious adverse events (SAE) non-COVID-19 cases, SAE COVID-19 cases and of adverse events of special interest (AESI), by dose of vaccine and study group.

Dose	Immunocompromised		Immunocompetent	
	SAE (n)	Nº of participants with SAE / Nº vaccinated (%)	SAE (n)	Nº of participants with SAE / Nº vaccinated (%)
**Non-COVID-19 cases**				
First	8	8 / 341 (2.3)	0	0
Second	17	13 / 237 (5.5)	2	2 / 100 (2.0)
Third	47^a^	24 / 230 (10.4)	0	0
Fourth	23	18 / 197 (9.1)	0	NA
**COVID-19 cases**				
First	3	3 / 341 (0.9)	0	0
Second	2	2 / 237 (0.8)	0	0
Third	7	7 / 230 (3.0)	0	0
Fourth	1	1 / 197 (0.5)	0	NA
**Dose**	**AESI (n)**	**Nº of participants with AESI / N**º **vaccinated (%)**	**AESI (n)**	**Nº of participants with AESI / N**º **vaccinated (%)**
First	3	3 / 341 (0.9)	0	0
Second	8	8 / 237 (3.0)	0	0
Third	7	6 / 230 (2.6)	1	1 / 100 (1.0)
Fourth	2	2 / 197 (0.5)	0	NA

NA = not applicable, received three doses; ^a^One participant received CoronaVac as a third dose.

**Table 5:** - Deaths during the study: participants, sex, age and chronic condition, number of COVID vaccine doses, date of the last dose, COVID status at the time of death date and cause of death.

Case	Sex	Age (years)	Chronic condition	Number of COVID vaccine doses^a^	Date of last dose	COVID status / Date	Time between last vaccine dose and death (days)	Date of death	Cause of death
1	Female	56	Kidney transplant	1	05/28/ 2021	**Positive** 06/09/2021	19	06/16/2021	COVID with respiratory failure
2	Female	45	Ovarian cancer	1	08/26/2021	Negative 09/14/2021	20	09/15/2021	Carcinomatosis with pleural effusion and multiple organ failure
3	Male	36	Colon cancer	3	10/15/2021	Negative 10/23/2021	28	11/12/2021	Lymphangitic and peritoneal carcinomatosis
4	Female	19	Kidney transplant	2	09/17/2021	Negative 11/12/2021	65	11/21/2021	Sacral osteomyelitis and sepsis
5	Female	23	Cancer (pleural mesothelioma)	3	11/05/2021	Negative 12/16/2021	47	12/22/2021	Cancer progression to pelvis (pubis, acetabulum) and spine, refractory to treatment
6	Female,	59	Cervical cancer	3	10/15/2021	Negative 12/11/2021	67	12/21/2021	Cancer progression with ascites and bile duct obstruction, refractory to treatment
7	Female	54	Lung transplant, O2-dependent	3	10/07/2021	**Positive** 01/28/2022	113	01/28/2022	COVID and severe bronchospasm
8	Female	49	Common variable immunodeficiency and cryptogenic cirrhosis	3^b^	09/29/2021	**Positive** 02/07/2022	140	02/16/2022	COVID with respiratory failure and cirrhosis decompensation
9	Female	30	Neuroblastoma	3	10/19/2021	Negative (six samples from 01/05 to 02/09/2022)	124	02/20/2022	Disease progression and respiratory failure due to alveolar bleeding
10	Male	52	Floor of mouth cancer with lung metastasis	3	01/06/2022	Negative 02/24/2022	79	03/26/2022	Tumor infection and bleeding, and progressive CNS disease
11	Male	45	Heart transplant	2^c^	08/05/2021	Negative 03/07/2022	217	03/10/2022	Congestive heart failure and bacterial pneumonia
12	Female	33	Heart transplant and dialytic chronic kidney disease	3	10/05/2021	Not performed	162	03/16/2022	Sudden death (cardiorespiratory arrest after dialysis)
13	Male	53	Glottis cancer	3	11/17/2021	Negative 03/25/2022	143	04/09/2022	Cancer progression
14	Male	49	Kidney transplant	4	01/31/2022	Not performed	87	04/28/2022	Respiratory failure, shock and renal failure
15	Female	36	Breast cancer with bone metastasis	4	02/22/2022	Negative 05/01/2022	68	05/01/2022	Cancer progression
16	Female	53	Kidney transplant	3	09/27/2021	Positive 05/12/2022	259	06/13/2022	COVID with kidney and respiratory failure
17	Male	46	Cancer (gastrointestinal stromal tumor), with metastasis	4	04/04/2022	Negative 08/09/2022	142	08/24/2022	Cancer progression, with lung, bones and peritoneum metastasis, refractory to treatment
18	Male	30	Heart transplant	4	02/14/2022	Negative 07/29/2022	169	08/02/2022	Heart failure
19	Male	54	Pancreatic head adenocarcinoma	4	03/15/2022	Negative 07/31/2022	138	07/31/2022	Cholangitis and sepsis

^a^First and second dose of CoronaVac, most patients received BNT162b2 (Pfizer) as the third and fourth dose; ^b^Only one patient received all three doses of CoronaVac; ^c^The patient had an episode of acute rejection 13 days after the second dose and did not take the third dose. He presented two episodes of acute rejection before COVID vaccination.

Table 4 and Supplementary Table S3 present 21 AESI in 19 participants (18 IC and one H) reported during the study. Four AESI were considered related to the vaccine: three cases of erythema multiforme in IC participants following CoronaVac (one case after the first dose and two cases after second dose) and one paresthesia in an immunocompetent participant, after mRNA162b2 [third dose]). All four cases were mild to moderate, started one day after vaccination and fully recovered.

## DISCUSSION

In our study, considering any adverse reactions after the first and second doses, no statistically significant differences in the frequency of solicited adverse reactions following the first and second CoronaVac doses were detected between IC and H participants. After the third dose of BNT162b2, greater frequency of AR was detected among the H group. Most AR following vaccination were mild or moderate in both immunocompromised and immunocompetent participants. The most serious AE were related to IC participants’ underlying conditions. Only one SAE was considered as possibly related to vaccination: a rejection in a heart transplant recipient was classified as temporally associated to vaccination, but no literature evidence was found to establish causal association (B1). Four adverse events of special interest (three erythema multiforme after CoronaVac in IC participants and one paresthesia after BNT162b2 in an H participant) were classified as related to the vaccines, all of them were mild and the participants fully recovered. In the literature, we found only one report of erythema multiform cases, five days after the second CoronaVac dose, in a Brazilian 75-year-old man^10^.

Few studies evaluated CoronaVac in immunocompromised hosts. A study conducted in Türkiye analyzed 89 immunosuppressed participants with psoriasis vaccinated with CoronaVac (n=44) or mRNA162b2 (n=45). The frequency of adverse events was lower than in our study. In the aforementioned study, 28% of immunocompromised participants presented local adverse events and 15.7% presented systemic adverse events^11^, whereas in our study, 62.6% of IC participants reported local pain after the third mRNA dose, and 31.1% had headaches after the first CoronaVac dose. However, the Turkish study collected information about AE three to six weeks after the second dose of each vaccine. This procedure could have led to underreporting of mild symptoms, explaining the difference.

Another study included 17 immunosuppressed patients with Systemic Lupus Erythematous (SLE), vaccinated with mRNA BNT162b2, in Israel^12^. The vaccine was well tolerated, with mild adverse events that included flu-like symptoms, fever, chills, weakness, malaise, headache, myalgia, arthralgia, and pain at injection site. No SLE flare was documented. Despite the small number of participants with rheumatic diseases in our study (n=21), no flare was reported.

An observational prospective study in Japan^13^ included 44 allogeneic HSCT recipients and 38 healthy individuals, who received mRNA BNT162b2 or mRNA-1273 (Moderna). All participants reported at least one symptom after the first and second doses. Similarly to our study, pain at the site was the most common adverse event. All symptoms were mild and participants recovered spontaneously. In our study, most solicited AR were mild, but there were some moderate and severe events (Supplementary Tables S1A, S1B, S1C and S1D) and all participants recovered.

A study in Poland evaluated 300 kidney transplant^14^ recipients who received mRNA BNT162b2 (Pfizer) or mRNA-1273 (Moderna), with most participants receiving the former (60%). The frequency of local AR was higher than in our study (84.7% after the first dose and 65.3% after the second dose). Most participants had mild symptoms, similar to our findings. Frequency of systemic solicited AR was also similar to our study, considering both vaccines. Another study, in China, included liver transplant adult recipients^15^ (n=35), who received a two-dose CoronaVac schedule. The vaccine was well tolerated and no serious adverse events or rejection was reported, unlike our study.

May et al. conducted a systematic review of COVID-19 vaccines booster doses in hematological and solid cancer patients, including 22 articles. Most reviewed studies evaluated mRNA vaccines by Pfizer (n=20) or Moderna (n=13); five evaluated vector-based vaccine by Janssen and two evaluated ChAdOx/AZ. Most studies did not report any adverse event^16^. One study reported mild AE in 26.8% of participants following mRNA BNT162b2; the most common were fatigue, weakness, myalgia and fever^17^. Another study that evaluated mRNA BNT162b2 and Ad26.COV2.S reported two severe (grade 3 or 4) events (fever and fatigue)^18^.

A limitation of our study was the small number of participants in each subgroup (SOT, HSCT, cancer, IEI, rheumatologic diseases), which made it not feasible to perform a subgroup analysis. However, our study included a large number of immunocompromised participants with different conditions, and there are few studies on safety of heterologous vaccination among this population.

## CONCLUSION

In this study, the heterologous schedule of two CoronaVac doses followed by mRNA BNT162b2 was safe in immunocompromised participants with different underlying conditions during the study follow-up (12 months). A booster dose of BNT162b2 did not significantly increase the frequency of adverse reactions.
