# Erratum

**DOI:** 10.1590/S1678-9946202567002err

**Published:** 2025-04-04

On the article "Safety of two-dose schedule of COVID-19 adsorbed inactivated vaccine (CoronaVac; Sinovac/Butantan) and heterologous additional doses of mRNA BNT162b2 (Pfizer/BioNTech) in immunocompromised and immunocompetent individuals", published in the Revista do Instituto de Medicina Tropical de São Paulo, Vol. 67, e2, 2025 (https://doi.org/10.1590/S1678-9946202567002):

Where it reads Maria Isabel de Moraes Pinto^7^ should be read Maria Isabel de Moraes Pinto^11^

